# Bis-sulfonic Acid Ionic Liquids for the Conversion of Fructose to 5-Hydroxymethyl-2-furfural

**DOI:** 10.3390/molecules171112804

**Published:** 2012-10-31

**Authors:** Sang Eun Sim, Sunjeong Kwon, Sangho Koo

**Affiliations:** 1 Department of Nano Science and Engineering, Myong Ji University, San 38-2, Nam-Dong, Yongin, Kyunggi-Do 449-728, Korea; Email: longberk@hanmail.net (S.E.S.); kksj1675@hanmail.net (S.K.); 2 Department of Chemistry, Myong Ji University, San 38-2, Nam-Dong, Yongin, Kyunggi-Do 449-728, Korea

**Keywords:** biomass conversion, D-fructose, 5-hydroxymethyl-2-furfural, acid ionic liquid, sulfonic acid

## Abstract

Homogenous bis-sulfonic acid ionic liquids (1 mol equiv.) in DMSO (10 mol equiv.) at 100 °C efficiently mediated the conversion of D-fructose into 5-hydroxymethyl-2-furfural in 75% isolated yield, which was roughly a 10% increment compared to the case of the mono-sulfonic acid ionic liquids.

## 1. Introduction

**Scheme 1:**
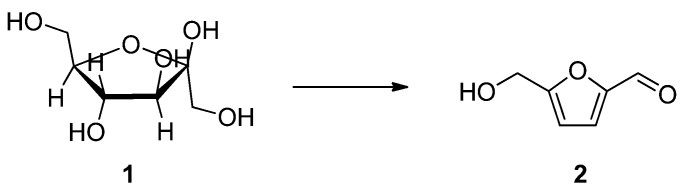
D-Fructose (**1**) conversion to 5-HMF (**2**).

Generating a sustainable energy source from the readily available natural resources is one of the most important and urgent challenges of mankind. Much attention has focused on the biomass conversion into the platform chemical such as 5-hydroxymethyl-2-furfural (5-HMF) (**2**) [1,2], which can be further transformed to bio-fuels or other commodity chemicals [3,4]. D-Fructose (**1**) is the basic and the most widely studied biomass, whereby dehydration with elimination of three water molecules directly produces 5-HMF (**2**) (Scheme 1) [5]. Biomass conversion in ionic liquids has been extensively studied due to their strong solvent power as well as good thermal stability, in which acids were normally added to the reaction media in order to facilitate the dehydration step [6,7,8,9,10,11,12,13,14].

We were interested in ionic liquids containing an acid moiety in the expectation of the synergistic effect on the acidity as well as the solubility [15]. There have been several reports on the biomass conversion into 5-HMF utilizing the ionic liquids containing mono-sulfonic acid as reaction media or as catalysts [16,17,18,19]. Sulfonic acids seemed to be suitable promoters for the dehydration of sugars due to their ideal p*K*_a_ values for the dehydration [20]. The preparation and measurement of the p*K*_a_ values of the bis-sulfonic acid ionic liquids has been recently published, which were efficiently utilized as catalysts in the esterification of glycerol and Fischer indole synthesis [21,22]. It appeared that the bis-sulfonic acid ionic liquids might as well be better promoters for the biomass conversion into 5-HMF, and we herein demonstrated the efficiency of the bis-sulfonic acid ionic liquids as efficient promoters for the fructose conversion into 5-HMF, together with the comparison with the mono-sulfonic acid ionic liquids.

## 2. Results and Discussion

**Scheme 2:**
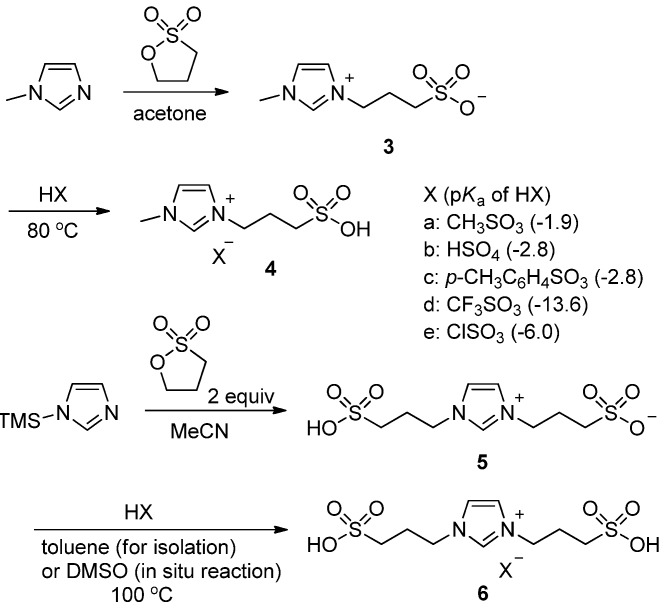
Preparation of mono- and bis-sulfonic acid ionic liquids **4** and **6**.

The zwitterionic salts **3** and **5** containing a sulfonate anion were prepared by the reaction of propanesultone with 1-methylimidazole and 1-(trimethylsilyl)imidazole, respectively [20,22]. Strong acids (HX) which were selected among the groups of methanesulfonic acid (p*K*_a_ −1.9), sulfuric acid (p*K*_a_ −2.8), *para*-toluenesulfonic acid (p*K*_a_ −2.8), trifluoromethanesulfonic acid (p*K*_a_ −13.6), and chlorosulfonic acid (p*K*_a_ −6.0) should be added to protonate the sulfonate anion, thereby forming the ionic liquids **4** and **6** containing mono- and bis-sulfonic acids, respectively (Scheme 2). The zwitterion **5** itself is an ionic liquid containing a sulfonic acid.

The ionic liquids **4** were easily prepared by heating the mixture of the acid and the salt **3** at 80 °C for 1~2 h. On the other hand, solvent (toluene or water) was required for the generation of the ionic liquids **6** due to their high melting points. It is much convenient to use toluene as a solvent to prepare the ionic liquids **6** as pure white solid forms, whereas a longer reaction time as well as thorough drying was required from the aqueous solutions [22]. When DMSO was used as a reaction medium for the fructose conversion, the isolation of the ionic liquids **6** was not necessary. The acid and the salt **5** in DMSO were heated at 100 °C for 2 h before fructose was added, which gave almost identical results with the cases where the isolated ionic liquids **6** were used in DMSO.

The effects of acid, acid ionic liquid, and solvent on the conversion of fructose to 5-HMF was examined first and the results are summarized in Table 1. The reaction was carried out at 100 °C for 1 h using 0.5 g of D-fructose. Methanesulfonic acid (MsOH) alone contributed little effect on the formation of 5-HMF regardless of the amount of acid added (entries 1–2). Ten mol equiv. of acid ionic liquid **4a** prepared by the addition of MsOH to the zwitterionic salt **3** produced 5-HMF in 31% yield (entry 4). The amount of acid ionic liquid **4a** can be reduced to 1 mol equiv. by the use of organic solvent, among which DMSO is better than any other solvent (entries 5–8). DMSO is known to be a favorable solvent for fructose dehydration due to its ability to prevent the formation of by-products, levulinic acid and humins, from the resulting 5-HMF [16]. On the other hand, water is conceived to have a negative effect on the dehydration of sugars to 5-HMF, because 5-HMF may react with water to generate levulinic acid and formic acid or furfural under acidic conditions [18,19,23,24]. Therefore, excessive water should be avoided from the reaction medium (entry 6). It is also important to use the right amount of solvent to give the optimum concentration (*ca.* 1.4 M solution) for the fructose conversion. A slight decrease in the yield of 5-HMF was noticed for diluted solutions (*ca.* 0.14 M and 0.28 M) of fructose in DMSO (entries 8–10). When one mol equiv. of acid ionic liquid **4a** is used for fructose (0.5 g), ten mol equiv. of DMSO (2 mL) provides the optimum concentration, in which 100% conversion of fructose and 66% yield of 5-HMF were obtained (entry 10).

**Table 1:** The effect of the acid ionic liquid **4a** and solvent on the conversion of D-fructose (**1**) into 5-HMF (**2**) ^a^.

Entry	3 (equiv.)	MsOH (equiv.)	Solvent (mL)	Conversion (%) ^b^	Yield 2 (%) ^c^	Selectivity (%)
1	-	1	EtOAc (10)	96.3	5.0	5.2
2	-	10	-	81.5	3.0	3.7
3	1	1	-	99.5	5.0	5.0
4	10	10	-	95.6	30.8	32.2
5	1	1	EtOAc (10)	99.6	41.0	41.2
6	1	1	H_2_O (10)	17	0.7	4.1
7	1	1	MeCN (10)	92.7	41.3	44.6
8	1	1	DMSO (10)	100	59.8	59.8
9	1	1	DMSO (20)	100	59.3	59.3
10	1	1	DMSO (2)	100	65.6	65.6

^a^ The reactions were carried out at 100 °C for 1 h using 0.5 g of D-fructose (1); ^b^ The conversion was calculated based on the recovered D-fructose in aqueous layer by HPLC analysis (2:8 H_2_O/MeCN; flow rate 1~2 mL/min; PDA detector at 195 nm) using the Waters Carbohydrate Analysis Column (3.9 × 300 mm); ^c^ The Yield (%) was calculated based on 5-HMF in organic layer by HPLC analysis (5:5 H_2_O/MeOH; flow rate 2 mL/min; PDA detector at 285 nm) using Watchers 120 ODS (4116B) column (4.6 × 250 mm).

The effects of the amount of acid ionic liquid, reaction temperature and time, and the types of sulfonic acid ionic liquids on the fructose conversion to 5-HMF were then studied systematically under the above solvent conditions (2 mL DMSO for 0.5 g D-fructose), and the results are summarized in Table 2. Since the dehydration of fructose was reported to proceed in high boiling DMSO even without acid catalysts, the blank experiment was carried out first [9]. D-Fructose in DMSO was heated to 100 °C for 1 h to produce 5-HMF in only 15% yield (entry 1). The mono-sulfonic acid ionic liquid **4a** was then selected again for the optimization study. The yield of 5-HMF was continuously increased as the amount of **4a** was increased up to one mol equiv., and then decreased back (entries 2–7). This phenomenon can be explained based on the following contrasting facts that sufficient active site from the ionic liquid should be available for the facile fructose dehydration and that the acidic ionic liquids promote degradation of 5-HMF at elevated temperature [9,18,20]. We set the optimum temperature and time as 100 °C and 1 h, even though almost identical result was obtained in 2 h at 100 °C (entries 8–11). However, humin and insoluble polymers were obtained after a prolonged reaction time.

**Table 2:** The conversion of *D*-fructose (**1**) into 5-HMF (**2**) under the acidic ionic liquids in DMSO ^a^.

Entry	Ionic liquid (equiv.)	Temp. (°C)	Time (h)	Yield 2 (%) ^b^
1	-	100	1	15
2	**4a** (0.1)	100	1	48
3	**4a** (0.3)	100	1	51
4	**4a** (0.5)	100	1	59
5	**4a** (0.7)	100	1	62
6	**4a** (1.0)	100	1	66
7	**4a** (2.0)	100	1	50
8	**4a** (1.0)	80	1	54
9	**4a** (1.0)	120	1	54
10	**4a** (1.0)	100	0.5	52
11	**4a** (1.0)	100	2	66
12	**4b** (1.0)	100	1	65
13	**4c** (1.0)	100	1	67
14	**4d** (1.0)	100	1	63
15	**4e** (1.0)	100	1	23
16	**5** (1.0)	100	1	57
17	**6a** (1.0)	100	1	75
18	**6b** (1.0)	100	1	72
19	**6c** (1.0)	100	1	64
20	**6d** (1.0)	100	1	55
21	**6e** (1.0)	100	1	33

^a^ The reactions were carried out using 0.5 g of D-fructose (1 equiv.) in 2 mL of DMSO; ^b^ Isolated yield after silica gel column chromatography.

The efficiencies of various mono- and bis-sulfonic acid ionic liquids for the fructose conversion to 5-HMF were then compared under the above optimized conditions (entries 12–21). The p*K*_a_ of the sulfonic acids (or the counter anions of the ionic liquids) did not make much differences in the product yield (~60% isolation of 5-HMF) for the mono-sulfonic acid ionic liquids **4a**–**d** except for the case of chlorosulfonic acid in **4e** (entries 12–15). The zwitterionic salt **5** can be regarded as a mono-sulfonic acid ionic liquid, in which the counter ion is tethered by the propylene bridge. An almost similar yield of 5-HMF (57%) was obtained for **5** as the other mono-sulfonic acid ionic liquids **4a**–**d** (entry 16). The bis-sulfonic acid ionic liquid **6e** prepared from the strong super acid was not effective in converting D-fructose into 5-HMF (entry 21) like in the case for the monosulfonic acid ionic liquid **4e**. It was reported that strong acids afford undesirable by-products such as humins. The mildest sulfonic acids, methanesulfonic acid and sulfuric acid, provided the best yield (~75%) of 5-HMF (entries 17–18). The homogeneity of the reaction media (the acid ionic liquid and DMSO) may be the most important issue in these bis-sulfonic acid ionic liquids. It is obvious that the bis-sulfonic acid ionic liquids **6** are generally better promoters than the mono-sulfonic acid ionic liquids **4** in converting fructose to 5-HMF.

## 3. Experimental

### 3.1. General

^1^H-NMR spectra were recorded on a Varian 400 MHz NMR spectrometer in deuterated water (D_2_O) for ionic liquids and deuterated chloroform (CDCl_3_) for 5-HMF. Solvents for extraction and chromatography were reagent grade and used as received. The column chromatography was performed by the method of Still with silica gel 60, 70–230 mesh ASTM supplied by Merck using a gradient mixture of EtOAc/hexanes. Reactions were performed in a flask open to the air unless noted otherwise. HPLC analysis for the sugar in aqueous layer was carried out using the Waters Carbohydrate Analysis Column (3.9 × 300 mm) with H_2_O/MeCN (2:8) as a mobile phase (flow rate 1~2 mL/min), while 5-HMF in organic layer was analyzed using Watchers 120 ODS (4116B) column (4.6 × 250 mm) with H_2_O/MeOH (5:5) as a mobile phase (flow rate 2 mL/min). PDA detector was used to measure each compound either at 195 nm (D-fructose) or at 285 nm (5-HMF).

### 3.2. Preparation of **3**

To a stirred solution of 1-methylimidazole (7.93 mL, 100 mmol) in acetone (100 mL) at 0 °C was added propanesultone (12.21 g, 100 mmol) in acetone (80 mL). The mixture was then warmed to and stirred at room temperature for 1 day. The crude product was filtered, rinsed thoroughly with acetone, dried under reduced pressure to give **3** (19.61 g, 96 mmol) in 96% yield as a white solid. ^1^H-NMR (D_2_O) δ 2.31 (m, 2H), 2.89–2.93 (m, 2H), 3.89 (s, 3H), 4.36 (t, *J* = 7.2 Hz, 2H), 7.44 (d, *J* = 2.0 Hz, 1H), 7.52 (d, *J* = 2.0 Hz, 1H) ppm.

### 3.3. Representative Procedure for the Preparation of **4a**

The imidazolium sulfonate **3** (0.30 g, 1.48 mmol) and methanesulfonic acid (0.10 mL, 1.48 mmol) were heated at 80 °C for 2 h. The homogeneous mixture was then cooled to room temperature, rinsed with Et_2_O, and concentrated under reduced pressure to give **4a** (0.43 g, 1.44 mmol) in 97% yield. ^1^H-NMR (D_2_O) δ 2.31 (m, 2H), 2.80 (s, 3H), 2.89–2.93 (m, 2H), 3.89 (s, 3H), 4.36 (t, *J* = 7.2 Hz, 2H), 7.44 (s, 1H), 7.52 (s, 1H), 8.75 (s, 1H) ppm.

### 3.4. Preparation of **5**

To a stirred solution of 1-(trimethylsilyl)imidazole (1.49 g, 10.6 mmol) in MeCN (20 mL) was added propanesultone (2.60 g, 21.25 mmol). The mixture was stirred at room temperature for 1 day. The crude product was filtered, rinsed thoroughly with acetone, and dried under reduced pressure to give **5** (2.95 g, 9.43 mmol) in 89% yield as a white solid. ^1^H-NMR (D_2_O) δ 2.33 (m, 4H), 2.89–2.95 (m, 4H), 4.38 (t, *J* = 7.2 Hz, 4H), 7.57 (s, 2H), 8.82 (s, 1H) ppm. 

### 3.5. Representative Procedure for the Preparation of **6a**

The imidazolium sulfonate **5** (0.87 g, 2.78 mmol) and methanesulfonic acid (0.18 mL, 2.78 mmol) in toluene (10 mL) were heated at 100 °C for 2 h. The homogeneous mixture was then cooled to room temperature and concentrated under reduced pressure. The crude product was rinsed with Et_2_O and then dried in a vacuum oven to give **6a** (1.10 g, 2.70 mmol) in 97% yield. ^1^H-NMR (D_2_O) δ 2.33 (m, 4H), 2.80 (s, 3H), 2.89–2.96 (m, 4H), 4.38 (t, *J* = 7.2 Hz, 4H), 7.57 (s, 2H), 8.82 (s, 1H) ppm.

### 3.6. Representative Procedure for 5-HMF (**2**) Using the Acid Ionic Liquid **6a**

The imidazolium sulfonate **5** (0.87g, 2.78 mmol) and methanesulfonic acid (0.18ml, 2.78mmol) were mixed in DMSO, and the mixture was heated at 100 °C for 2 h. D-Fructose (0.5 g, 2.78 mmol) was then added to the mixture, and heated for 1 h. The color of the mixture turned to dark brown. The mixture was cooled to room temperature, quenched with water, extracted with EtOAc, dried over sodium sulfate, filtered, and concentrated under reduced pressure. The crude product was purified by SiO_2_ flash column chromatography to give 5-HMF (0.26 g, 2.08 mmol) in 75% yield. ^1^H-NMR (CDCl_3_) δ 2.24 (br s, 1H), 4.73 (s, 2H), 6.52 (s, 1H), 7.22 (s, 1H), 9.61 (s, 1H) ppm.

## 4. Conclusions

We demonstrated that the homogenous bis-sulfonic acid ionic liquids **6** are efficient mediators for the conversion of D-fructose to 5-HMF. Up to 75% yield of 5-HMF was isolated in DMSO at 100 °C for 1 h using the bis-sulfonic acid ionic liquid **6a** prepared by the addition of methanesulfonic acid to the zwitterionic salt **5**, which was ~10% higher than the yield obtained from the use of the mono-sulfonic acid ionic liquid **4a**. Studies on the other biomass (e.g., glucose and cellulose) conversion to 5-HMF and further conversions to the other useful chemicals using the bis-sulfonic acid ionic liquids **6a**–**b** are currently underway together with an effort for efficient recycling of the ionic liquids from the highly polar and high boiling DMSO solution.
